# Application and Molecular Mechanisms of Extracellular Vesicles Derived from Mesenchymal Stem Cells in Osteoporosis

**DOI:** 10.3390/cimb44120433

**Published:** 2022-12-15

**Authors:** Yajing Yang, Lei Yuan, Hong Cao, Jianmin Guo, Xuchang Zhou, Zhipeng Zeng

**Affiliations:** 1 School of Sport Medicine and Rehabilitation, Beijing Sport University, Beijing 100084, China; 2 Cancer Hospital & Shenzhen Hospital, Chinese Academy of Medical Sciences and Peking Union Medical College, Shenzhen 518116, China; 3 School of Kinesiology, Shanghai University of Sport, Shanghai 200438, China

**Keywords:** osteoporosis, extracellular vesicles, exosomes, miRNAs, mesenchymal stem cells

## Abstract

Osteoporosis (OP) is a chronic bone disease characterized by decreased bone mass, destroyed bone microstructure, and increased bone fragility. Accumulative evidence shows that extracellular vesicles (EVs) derived from mesenchymal stem cells (MSCs) (MSC-EVs), especially exosomes (Exos), exhibit great potential in the treatment of OP. However, the research on MSC-EVs in the treatment of OP is still in the initial stage. The potential mechanism has not been fully clarified. Therefore, by reviewing the relevant literature of MSC-EVs and OP in recent years, we summarized the latest application of bone targeted MSC-EVs in the treatment of OP and further elaborated the potential mechanism of MSC-EVs in regulating bone formation, bone resorption, bone angiogenesis, and immune regulation through internal bioactive molecules to alleviate OP, providing a theoretical basis for the related research of MSC-EVs in the treatment of OP.

## 1. Introduction

Osteoporosis (OP) is a systemic bone disease characterized by weakened bone structure strength, decreased bone mass, and increased fracture risk [1]. Due to the high rate of disability, morbidity, and mortality, OP causes a heavy burden to patients’ families and society, which is a major public health problem [2]. OP can be divided into primary OP and secondary OP. Primary OP is a systemic skeletal disease closely related to postmenopausal estrogen deficiency or age [3], while secondary OP refers to adverse reactions to drugs, changes in physical activity, or other diseases, such as glucocorticoids and restricted activity [4]. Under physiological and pathological conditions such as estrogen deficiency, aging, disuse, drug, and malnutrition, bone formation and bone absorption are decoupled or/and osteoblast differentiation and adipocyte differentiation of bone marrow mesenchymal stem cells (BMSCs) are unbalanced, which often leads to the occurrence and development of OP [5]. Therefore, coordinating osteoclastic–blastic coupling or osteo-adipogenic balance is an effective way to prevent and treat OP.

As an important member of the stem cell family, MSCs not only have the potential of self-renewal and multi-directional differentiation, including osteoblasts, chondrocytes, and adipocytes, but also have the characteristics of promoting angiogenesis, anti-inflammation, anti-apoptosis, and immune regulation. Therefore, MSCs are the most commonly used cell type in tissue engineering strategies [6]. Cumulative studies have shown that MSCs change the microenvironment of damaged tissues and repair damaged tissues mainly through paracrine action, rather than self-proliferation and differentiation to replace damaged tissues [7,8,9]. However, MSCs-based cell therapy still has some limitations, including invasive collection, a small amount of isolated cells, age dependency [10], immune rejection, and acquired gene mutation [11]. Recent studies have found that extracellular vesicles (EVs) are the key paracrine factors released by MSCs [12]. MSC-EVs contain many nucleic acids, proteins, lipids, and genetic molecules from parent cells, such as messenger RNAs (mRNAs), microRNAs (miRNAs), and long non-coding RNAs (lncRNAs). MSC-EVs can mediate intercellular communication and regulate the cellular behavior of receptor cells by delivering encapsulated bioactive components [13]. In addition, MSC-EVs also have therapeutic functions similar to those of their parent stem cells, such as repairing damaged tissues, inhibiting inflammatory response, and regulating immune response [14]. Compared with MSCs transplantation, the cell-free therapy based on MSC-EVs has the advantages of low immunogenicity, non-tumorigenicity, non-vascular thrombosis, easy preservation, easy acquisition, and transformation [15]. Moreover, the unique lipid bilayer structure of EVs ensures stable cargo transport and protects biological molecules such as RNA from rapid degradation [16]. Importantly, MSC-EVs can be manipulated and genetically modified to improve the delivery efficiency of bioactive molecules [17]. In recent years, MSC-EVs-based strategies have been extensively studied in tissue regeneration and disease treatment [18]. BMSC-EVs overexpressing mutant hypoxia inducible factor-1α (HIF-1α) enhanced the therapeutic effect of steroid-induced femoral head necrosis in rabbit models [19]; BMSC-EVs can promote fracture healing [20]. The above research lay a foundation for the research on the prevention and treatment of OP by MSC-EVs. Although preliminary studies have shown that the cell-free therapy based on MSC-EVs is a promising therapeutic strategy for OP [21,22,23,24,25]; however, the research on MSC-EVs in treating OP is still in its early stage. Deeply understanding the potential mechanism of MSC-EVs regulating OP contributes to formulating more effective treatment strategies. By reviewing the relevant research literature on MSC-EVs and OP, we summarized the bone targeting effect of engineered MSC-EVs in OP and the potential mechanism of MSC-EVs regulating OP, providing a basis for clinical application research of MSC-EVs in OP.

## 2. Overview of EVs

**Figure 1:**
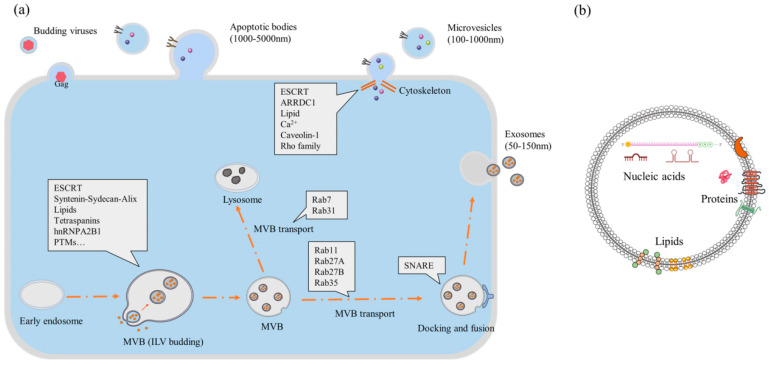
The biogenesis of the extracellular vesicle and its structure. (**a**) EVs can be divided into three subtypes: exosomes, microvesicles, and apoptotic bodies. (**b**) The extracellular vesicle is made up of lipid bilayers and enriched in proteins, nucleic acids, and lipids (The figure is cited from Jin Y, Ma L, Zhang W, Yang W, Feng Q, Wang H. Extracellular signals regulate the biogenesis of extracellular vesicles [34]).

EVs are nanoscale membrane vesicles with double membrane structure secreted by cells. Since it was first discovered in 1964, EVs have been found to be the carrier of long-distance signal transmission between cells, which may be rich and stable disease biomarkers in blood circulation [26]. EVs regulate target cell functions mainly through three ways: (1) EVs can directly combine with signal molecules on the cell membrane to regulate downstream signals; (2) EVs can fuse with the cell membrane to transfer bioactive substances to the cytoplasm, thereby activating or deactivating the relevant signal pathways in cells; (3) EVs can enter cells through endocytosis and release their contents to target organelles [27]. Therefore, EVs are considered as a good intercellular “communicator”. EVs can be divided into three categories according to their diameters, components, and sources: (1) Apoptotic bodies: apoptotic bodies are produced by shedding from cells when apoptosis occurs; (2) Microvesicles (MVs): MVs are produced by directly budding from the plasma membrane. The production process of MVs is related to the increase of intracellular calcium concentration and the remodeling of the membrane skeleton; (3) Exosomes (Exos): Exos are secreted by polycystic bodies and released mainly through exocytosis, which is related to the activation of cytoskeleton and calcium concentration [28] (as shown in Figure 1). Among them, Exos are the most attractive EV category. In 1983, a small membranous vesicle released from mature reticulocytes of sheep was reported for the first time, which was initially considered as the “waste” discharged by cells and was named Exos [29]. However, in recent years, studies have shown that Exos, as mediators of intercellular crosstalk, can participate in the communication between cells and exhibit specific biological effects while transferring functional active substances. Exos are nanoscale (30–150 nm in diameter) multi-vesicles that are spontaneously generated and actively secreted by a variety of active cells in mammals [30]. Exos are the products formed by the fusion of intraluminal vesicles derived from multi-vesicular bodies with the plasma membrane and released to the outside of the cell, which can be produced under normal or pathological conditions [31]. Multi-vesicles can fuse with lysosomes to degrade their contents, or fuse with plasma membranes to release inner membrane vesicles to the surrounding extracellular matrix, forming Exos containing a variety of bioactive substances [32]. Up to now, more than 4600 bioactive substances including proteins and nucleic acids have been found in Exos, such as dsDNA, miRNAs, major histocompatibility complex I, and heat shock protein HSC7023, which can widely participate in the regulation of various physiological and pathological processes in the body, such as antigen presentation, immune response, genetic material exchange, angiogenesis, inflammatory response, and tumor metastasis, thus playing an important role in the treatment of various diseases, such as the complex graft versus host diseases, myocardial ischemia and reperfusion injury, acute renal injury, and neurodegenerative diseases [33].

EVs have been widely studied and applied in tissue repair and regeneration due to their advantages of high circulatory stability, low immune rejection, good targeting, and regulation of receptor cell behavior [35]. In particular, EVs derived from mesenchymal stem cells (MSC-EVs) have the characteristics of low toxicity, high biocompatibility, and biological permeability, showing high potential in the development of drug delivery strategies. Numerous studies have shown that mesenchymal stem cell derived EVs (MSC-EVs) have similar therapeutic effects in tissue repair with their parent cells, such as reducing myocardial fibrosis [36], promoting wound healing [37], and cartilage regeneration [14]. In addition, it has been reported that MSC-EVs can induce new bone formation by enhancing osteoblast activity and promoting angiogenesis [38,39]. Other studies have shown that systemic application of MSC-EVs can alleviate the OP phenotype and stimulate bone regeneration [5,40], which highlights the potential of MSC-EVs in the treatment of bone diseases, especially OP.

## 3. Application of Bone-Targeted MSC-EVs in OP

Previous studies have shown that local application of MSC-EVs can induce bone formation and repair bone defects in bone defect models [39,41,42]. However, OP is a systemic bone metabolic disease. Only systemic administration, rather than local administration, can improve OP. Systemic administration of MSC-EVs can induce new bone formation by enhancing the function of osteoblasts, inhibiting the activity of osteoclasts, and promoting angiogenesis, thereby alleviating the OP phenotype in ovariectomy (OVX) mouse models, which highlights the potential of MSC-EVs in the treatment of OP [43]. However, unmodified MSC-EVs only showed limited therapeutic efficiency [44]. Lu et al. [45] showed that compared with the control group, the cortical bone mass of OP mice treated with BMSC-Exos injected via tail vein did not increase significantly. Recent studies have shown that most Exos administered systemically accumulate rapidly in the liver and spleen, but hardly in bone tissue. Short circulation time inhibits their targeting to pathological tissues and limits their potential as effective therapeutic drugs [46], which may partly explain why BMSC-Exos administered systemically has poor specific targeting performance to bone tissue and cannot effectively promote the increase of bone mass. Therefore, improving the ability of MSC-EVs to specifically target bone through genetic engineering or other methods is a key research direction in OP management based on MSC-EVs. Currently, research on MSC-EVs with specific bone targeting ability have made some progress in promoting osteogenic differentiation and inhibiting osteoclast differentiation.

### 3.1. Bone Targeted MSC-EVs Promote Osteogenic Differentiation in OP

The clinical treatment of OP mainly depends on anti-resorption drugs. However, a basic common disadvantage of these drugs is that they cannot restore the lost bone mass but prevent bone absorption [47]. Compared with anti-bone resorption drugs, bone anabolic agents such as parathyroid hormone (PTH) and Evenity can induce bone formation. However, both of them have distinct disadvantages: PTH requires long-term continuous use (at least 2 years), because sudden termination of use will lead to a rapid decline in bone mineral density; Evenity may increase the risk of myocardial infarction, stroke, and cardiovascular death [48,49]. Therefore, it is urgent to develop new innovative treatment strategies based on promoting bone regeneration in OP patients. At present, most of the approved drugs for clinical treatment have poor selectivity to pathological tissues, which reduces their effectiveness and safety. The tissue-specific targeting can improve the bioavailability and toxicity of therapeutic drugs. Adapters are single-stranded DNA/RNA oligonucleotides that can bind to target molecules with high affinity and specificity in a three-dimensional structure [50]. Previous studies have confirmed that aptamers can target and identify pathological tissues, and specifically attack pathological tissues with covalently or physically aligned therapeutic compounds [51]. Aptamer functionalized drug loaded Exos have been reported to efficiently deliver molecular drugs/fluorophores to tumor cells, providing a promising delivery platform for cancer treatment [52]. According to these characteristics of aptamers, some scholars have constructed an aptamer functionalized BMSC-Exos (BMSC-Exo-Apt) prepared by conjugating BMSC specific aptamers with BMSC-Exos through group modification [53]. The 5′-UTR of the aptamer can be modified by an aldehyde group to form a stable Schiff base with the amino reaction of Exos membrane protein. This specific aptamer can significantly promote the internalization of BMSC-Exos into BMSCs in vitro. Furthermore, Luo et al. [54] applied BMSC-Exo-Apt complex to OP mice after OVX to verify whether Exos can preferentially accumulate in bone tissue to promote bone regeneration. OP mice were injected with BMSC-Exos-Apt intravenously once a week for two months. The results showed that compared with the unmodified BMSC-Exos group, BMSC-Exos-Apt treatment could effectively increase the bone mass of OVX mice by promoting osteogenesis [54], indicating that BMSC targeting aptamer functionalized BMSC-Exos could target bone tissue to avoid rapid metabolism and clearance, thus promoting BMSC-Exos induced bone regeneration. This study proposed an aptamer-based bone-specific targeting method for Exos delivery. The effect of the BMSC-Exo-Apt complex represents a new promising treatment strategy for OP. In addition, surface modification of Exos by introducing specific receptors or ligands is another potential strategy to regulate the distribution characteristics of Exos in vivo. Stromal cell derived factor 1 (SDF1) is a ligand of C-X-C motor chemokine receptor 4 (CXCR4) expressed mainly by BMSCs [55]. The high level of SDF1 in bone marrow can recruit CXCR4+ peripheral HSC for homing, while the up-regulation of CXCR4 expression in hematopoietic stem cells can enhance the homing of hematopoietic stem cells [56]. Therefore, SDF1/CXCR4 axis plays a key role in the transport of hematopoietic stem cells and the homing of BMSCs. According to this characteristic, Hu et al. [56] constructed highly expressed CXCR4 on the Exos surface derived from genetically engineered NIH-3T3 cells (mouse embryonic fibroblast cell line), which has the characteristic of specifically targeting bone. Increased miR-188 was found in BMSCs from elderly patients. Knockout of miR-188 inhibits adipogenic differentiation of elderly BMSCs, suggesting that targeting miR-188 can be used as a therapeutic means to promote bone formation. Hu et al. [57] fused the constructed Exos with liposomes carrying antagomiR-188 to obtain hybrid nanoparticles (NPs) with bone targeting and anti-miR-188 capabilities. The team further injected 18-month-old male mice with hybrid NPs, antagomiR-188, or antagomiR-188-loaded hybrid NPs intravenously every week for 8 weeks. The results showed that, compared with the other two groups, antagomiR-188-loaded hybrid NPs significantly reversed the age-related bone trabecular loss and reduced cortical bone porosity by inhibiting fat production and promoting osteogenesis of BMSCs in aged mice, showing significantly higher bone mass retention. It is suggested that this study provides a bone targeted RNA interference delivery strategy based on Exos modification, which is a promising anabolic therapy for age-related bone loss. In addition, alendronate (Ale) is the most widely used first-line anti-OP drug containing a P-C-P group of the pyrophate analog, which inhibits bone absorption by combining with hydroxyapatite on the bone surface with high affinity and strength [58]. However, the drug may cause side effects such as esophagitis and gastrointestinal discomfort [59]. Nanocarriers as drug delivery systems can prolong the drug circulation time of Ale, thereby reducing the dosage and side effects of drugs. Although Ale is a kind of bone targeting drug molecules, it is difficult to couple it to the drug carrier surface under normal conditions. As an efficient and convenient chemoselective conjunction method under mild conditions, “click chemistry” can solve this problem [60]. As we all know, the discoverer of “click chemistry” won the Nobel Prize in 2022. “Click Chemistry” has mild reaction, simple operation, easy purification, and no harmful by-products. It is widely used in the synthesis of functional polymers and cell markers [61]. Wang et al. [62] constructed an Ale-MSC-EVs complex by coupling an azide (N3) group modified Ale molecule and an alkynyl (DBCO) group modified MSC-EVs through “click chemistry”. Subsequently, the team evaluated the bone regeneration capacity of the Ale-MSC-EVs complex. It was found that Ale-MSC-EVs was well-tolerated without side effects. Ale-MSC-EVs can not only promote the proliferation and differentiation of BMSCs in vitro, but also effectively prevent OVX induced bone loss in OP rats, suggesting that Ale-MSC-EVs with specific bone targeting ability is a potential therapeutic strategy for OP. It also shows that the covalent binding method of “click chemistry” provides a new insight into for targeting ligand modification of EVs surface film, which can be used to improve the specific bone targeting ability of EVs and has broad application prospects.

In summary, the specific bone targeting ability of MSC-EVs modified by genetic engineering techniques with aptamers, ligands, or drugs is significantly enhanced compared to natural MSC-EVs. Although the exploration of bone-targeted MSC-EVs is still in its initial stages, the great potential of this direction of research in improving osteogenic differentiation points to new directions for further research in the clinical treatment of OP. In addition, despite the ability of Ale to confer bone targeting to EVs, it remains to be further investigated whether Ale-EVs have an inhibitory effect on osteoclasts. Moreover, since Ale-EVs act as a bone target delivery system, future studies may also try to load other drugs such as nucleic acid drugs, small molecule drugs, etc. into the targeted drug delivery system for combined treatment of OP.

### 3.2. Bone Targeted MSC-EVs Inhibit Osteoclast Differentiation in OP

Inhibition of osteoclast activation and differentiation is an effective strategy for blocking bone resorption. Osteoclast inhibitors (e.g., oestrogen, denosumab, and calcitonin) have been used in the clinic for many years. However, not only do these drugs fail to restore osteoblast function and reconstruct bone microarchitecture, but they may also have toxic effects on the circulatory system [3]. This side effect is largely attributed to their inability to accumulate in the bone microenvironment and act on the target cells [63]. Therefore, there is a need to develop a new specific targeted therapeutic approach with low toxicity and reversal of the OP process. Previous studies have shown that biomaterials can influence the behavior and intercellular activity of a single cell type [64]. Bioactive glass is a bioactive material with excellent bioactivity and biocompatibility, which has the ability to specifically target and repair bone. Active ions eluting from bioactive glass can activate downstream genes and signals [65]. It was shown that SrBG-eluted ions inhibit osteoclast differentiation [66]. LncRNA NRON, an ncRNA inhibitor, activates the transcription factor of nuclear factor of activated T cells (NFAT), and nuclear translocation of NFATc1 is an important component of the osteoblast differentiation process [67]. Yang et al. [68] used bioactive glass nanoparticles (BGN) to induce BMSCs to secrete heterogeneous EVs and constructed BGN-BMSC-EVs complex overexpressing lncRNA NRON. The results revealed that BGN-induced BMSC-EVs were specific in their ability to inhibit osteoblast differentiation in vitro, compared with BMSC-EVs. Further studies showed that BGN-BMSC-EVs significantly reduced bone loss in OP mice and improved biochemical indices of bone metabolism in peripheral blood with good biosafety and no biotoxicity, suggesting that BGN-BMSC-EVs may be a potential bone-targeting agent for OP. In addition, a new drug delivery system developed by loading RNA cargos into Exos through electroporation and membrane-anchored modifications could also be applied to specific bone-targeted therapies for OP [69]. Schnurri-3 (SHN3), encoded by the Hivep3 gene (also known as SHN3 gene), is a multifunctional protein that not only enhances osteoclast activity by promoting nuclear factor-κB ligand (RANKL) expression [70], but also inhibits osteogenic differentiation and reduces the formation of H-type vessels [71,72]. Cui et al. [73] constructed an engineered Exos delivery system BT-Exo-siShn3 by immobilizing the bone-targeting peptide modified with a diacyllipid tail on the Exos membrane via hydrophobic interaction and then loading the siRNA of Shn3 by electroporation. To improve the bone-targeting capacity of BT-Exo-siShn3, Exos of the BT-Exo-siShn3 were conjugated with the peptide SDSSD (Ser, Asp, Ser, Ser, Asp) through a diacyllipid insertion method, which conferred BT-Exo-siShn3 to deliver siRNA specifically to osteoblasts, thereby mediating osteoblast Shn3 gene silencing. BT-Exo-siShn3 was found to take advantage of the inherent anti-OP functional advantage of induced pluripotent stem cells induced MSCs (iMSC)-derived Exos, while cooperating with the siRNA of shn3 to play a role in specifically targeting osteoblasts and thus inhibiting osteoclast formation in vitro and in vivo. Compared to the unmodified exosome group, mice in the BT-Exo-siShn3 group showed improved bone microarchitecture, including increased bone trabecular volume, increased number of trabeculae, and decreased trabecular separation. Compared to the unmodified Exos group, mice in the BT-Exo-siShn3 group showed improved bone microarchitecture, including increased bone trabecular volume, increased number of trabeculae, and decreased trabecular separation [73]. The above results indicate bone-specific targeted delivery is of great significance in the treatment of OP. This bone-targeted engineered Exos delivery system is a feasible, efficient, and cell-free therapeutic option for the treatment of OP, which may also provide new ideas for the targeted treatment of other bone diseases.

In summary, natural MSC-EVs do not possess the property of specifically targeting bone tissue, which largely limits their potential as an effective drug strategy. The ability to confer specific targeting to MSC-EVs by binding to bioactive materials or by peptide modification shows positive promise in inhibiting osteoclast function in OP therapy-related studies. However, in contrast to these results, Xu et al. [74] found that MSC-EVs could promote osteoblast differentiation, suggesting that different cargoes in MSC-EVs may play completely opposite roles in osteoclast differentiation. Notably, the bone-targeted engineered Exos delivery system BT-Exo-siShn3 simultaneously has multiple functions of enhancing osteogenic differentiation, promoting H-type angiogenesis, and inhibiting osteoclast formation, although the complexity and technical challenges it currently involves may limit its clinical translation. It may be further developed after overcoming technical regulation and cost issues.

## 4. Mechanisms of MSC-EVs in OP

**Figure 2:**
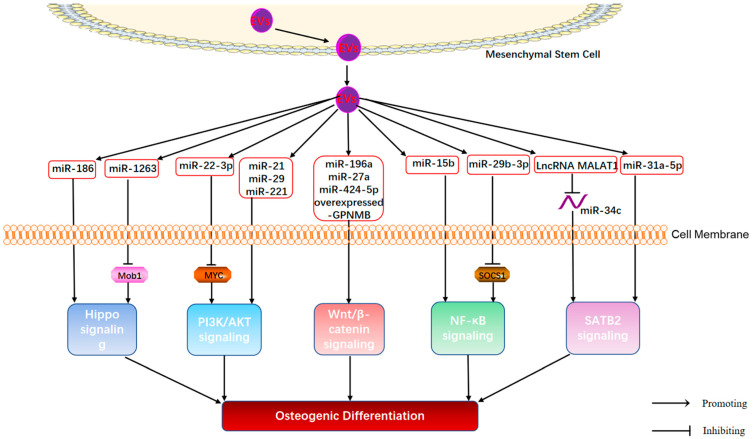
Mechanisms of MSC-EVs regulating the osteogenic differentiation in OP.

Cumulative studies have shown that MSC-EVs can influence downstream signaling cascades by directly translocating their internal cargoes, which in turn regulate bone formation, bone resorption, bone angiogenesis, and immune activity in OP [75,76] (as shown in Figure 2). There are many endogenous molecules encapsulated within MSC-EVs. Among them, miRNAs, one of the major bioactive substances in EVs, are the most attractive bioactive molecules [77]. miRNAs are small endogenous non-coding single-stranded RNAs of 18~25 nucleotides in length [78] that can induce changes in a variety of cellular processes, including cell proliferation, differentiation, senescence, and apoptosis [79]. However, the instability of miRNAs in the extracellular environment limits their application. EVs act as a special membrane vesicle capable of protecting miRNAs from degradation and delivering miRNAs to target cells, thus regulating cell-cell communication. Accumulating evidence suggests that MSC-EV-miRNAs play an important role in the treatment of OP. MiR-935, miR-21-5p, miR-27a-5p [24,25,80], miR-31a-5p [81], miR-29a [45], miR-146a [82] encapsulated in MSC-EVs were shown to be involved in the regulation of bone formation, bone resorption, angiogenesis, and immunomodulatory effect. An in-depth understanding of the specific mechanisms of MSC-EVs in OP would be beneficial to facilitate clinical translational research on MSC-EVs in OP.

### 4.1. Regulation of Bone Formation by MSC-EVs in OP

Bone remodeling consists of two processes: osteoblast-mediated bone formation and osteoclast-mediated bone resorption. Disruption of the homeostasis of bone remodeling may lead to OP. There is growing evidence that the crosstalk between monocyte-macrophage-osteoclasts and MSC-osteoblasts plays a crucial role in the pathological changes of OP [83]. Osteoblasts are derived from the osteogenic differentiation of MSCs [84], which is influenced by many environmental factors, such as hormones and growth factors [85]. A previous study confirmed the critical role of human bone marrow mesenchymal stem cell (hBMSC) osteogenic differentiation for bone regeneration therapy and the bone regenerative potential of hBMSC-EVs [86]. Importantly, human umbilical cord MSCs (HucMSCs)-derived Exos significantly promoted osteoblast differentiation and showed therapeutic effects in OVX mice [87]. Multiple bone formation-related signaling pathways have been identified to mediate the regulation of OP by MSC-Exos, such as Wnt/β-catenin, Hippo, phosphoinositide 3-kinase (PI3K)/Akt, nuclear factor kappa-B (NF-κB), and special AT-rich sequence-binding protein 2 (SATB2). Further understanding of the underlying mechanisms of osteogenic differentiation is essential for the development of effective therapeutic strategies for OP.

#### 4.1.1. Wnt/β-Catenin Signaling Mediates MSC-EVs Regulating Bone Formation in OP

Previous studies have confirmed that the Wnt signaling pathway can regulate the bone remodeling process by affecting both bone formation and bone resorption, thus participating in the initiation and development of OP [43]. The Wnt signaling pathway includes both classical and non-classical pathways. β-catenin protein, as a key signaling factor in the classical Wnt pathway, stimulates osteoblast differentiation and proliferative activity, which is most closely related to bone metabolism [88]. The classical Wnt/β-catenin signaling pathway mainly includes: ligand (Wnt extracellular protein), frizzled transmembrane receptor (Frizzled), receptor-related protein 5/6 (LRP-5/6), glycogen synthase kinase-3β (GSK-3β), and Axin. Studies have shown that the Wnt/β-catenin signaling pathway enhances osteoblast activity mainly by regulating the expression of osteogenic differentiation-specific genes, thereby promoting extracellular matrix mineralization to enhance bone formation and regulate bone remodeling [89]. Recent studies have demonstrated that MSC-EVs can promote osteoblast proliferation and differentiation, which may be closely related to Wnt/β-catenin signaling. Gong et al. [90] demonstrated the positive role of human embryonic stem cell-derived EVs (hESC-EVs) in reversing the senescence of BMSCs and promoting the proliferation and osteogenic differentiation potential of BMSCs through the transfer of encapsulated proteins. Bioinformatics analysis further revealed that protein components in hESC-EVs activate several classical signaling pathways involved in alleviating cellular senescence and promoting osteogenesis, including Wnt/β-catenin, which are involved in regulating the expression of anti-senescence genes to ameliorate MSCs senescence and promote osteogenic differentiation, either through direct or indirect interactions, or through synergistic interactions. It is suggested that Wnt/β-catenin signaling pathway may be involved in the regulatory role of EVs on OP. Unfortunately, this study did not further validate this. Since then, many scholars have further validated the role of Wnt/β-catenin signaling pathway in OP. Peng et al. [91] reported that BMSC-Exos can deliver the intrinsic miR-196a targeting to inhibit dickkopf-1 (DKK1), a negative regulator of the Wnt/β-catenin signaling pathway, to activate the Wnt/β-catenin signaling pathway, ultimately promoting osteogenic differentiation. In addition, miR-27, considered a key mediator of osteoblast differentiation, could promote reosseointegration in the regenerative treatment of peri-implantitis by directly targeting DKK2 [92]. Wang et al. [93] found that MSC-EVs had a similar protective effect on OP as miR-27a, while miR-27a inhibitor partially reversed the protective effect of MSC-EVs on OP. In contrast, knockdown of DKK2 reversed the inhibitory effect of miR-27a inhibitors on OP. Further studies revealed that miR-27a released from MSC-EVs positively regulated bone formation through the DKK2/Wnt/β-catenin signaling pathway, thereby effectively preventing OP in mice [93], although the above studies illustrate that unmodified MSC-EVs promote osteogenic differentiation through the Wnt/β-catenin signaling pathway. However, unmodified MSC-EVs showed limited therapeutic effects on OP. Previous studies have reported glycoprotein non-melanoma clone B (GPNMB) as a multifunctional transmembrane glycoprotein that plays a key role in osteoblast differentiation and bone homeostasis [94]. Huang et al. [95] explored the respective effects on OP by constructing BMSC-EVs overexpressing GPNMB compared to unmodified BMSC-EVs. The team found that GPNMB-modified BMSC-EVs were more effective in treating OP than unmodified BMSC-EVs. Mechanistically, BMSC-EVs significantly promoted the proliferation and osteogenic differentiation of BMSCs and reduced bone loss by activating the Wnt/β-catenin signaling pathway. In addition, studies have also identified a negative role of Wnt/β-catenin signaling in the treatment of OP by MSC-EVs. MiR-424-5p, one of the core miRNAs for tension-induced bone formation, is associated with pathological changes in osteosclerosis [96]. The Wnt inhibitor WIF-1 is a member of the Wnt protein secretion regulators that interacts directly with various Wnt ligands and attenuates their binding to membrane-bound receptors [97]. Wei et al. [98] revealed that BMSC-Exos overexpressing miR-424-5p inhibited osteogenic differentiation by suppressing the WIF1/Wnt/β-catenin signaling axis, while WIF1 overexpression partially reversed the osteogenic inhibitory effect of BMSC-Exos, indicating that interfering with miRNAs in MSC-Exos may be a new direction for the treatment of OP.

In summary, the Wnt/β-catenin signaling pathway is one of the important mechanisms involved in the treatment of MSC-EVs in OP. The promotion or inhibition of bone formation by the Wnt/β-catenin signaling pathway is influenced by the modification of MSC-EVs or the type of miRNAs in MSC-EVs. In addition to miRNAs, MSC-EVs contain many proteins, lipids, and other types of nucleic acids. Therefore, other intrinsically bioactive molecules derived from MSC-EVs, such as lncRNAs and circular RNAs (circRNAs), may also regulate OP through the Wnt/β-catenin signaling pathway, which requires further exploration.

#### 4.1.2. Hippo Signaling Mediates MSC-EVs Regulating Bone Formation in OP

Hippo signaling, consisting of an articulated protein and an inhibitory kinase, was first identified in Drosophila. Hippo signaling is a highly conserved signaling pathway between Drosophila and mammals [99]. The classical Hippo signaling pathway is functioned by the kinases MST1/2 and LATS1/2 that phosphorylate with SAV1 and Mob1 and inhibit the transcriptional coactivators YAP and TAZ [100]. It was found that the Hippo signaling pathway induces osteogenic differentiation of MSCs by upregulating the expression of runt-related transcription factor 2 (Runx2), alkaline phosphatase (ALP), and Osterix through the binding of the WW structural domain of TAZ to the PY motif of Runx2 [101]. Recent studies have found that Hippo signaling may also play a role in the treatment of OP by MSC-EVs. Yang et al. [102] explored the role of HucMSC-Exos in the regulation of proliferation and apoptosis of BMSCs in a rat model of disuse OP (DOP). This study found that in DOP, the expression of Mob1 was upregulated, which inhibited the activation of YAP and activated the Hippo signaling pathway. Interestingly, miRNA-1263 can inhibit Mob1 expression to reactivate the repressed YAP and directly impede the Hippo signaling pathway, thereby inhibiting BMSC apoptosis and promoting osteogenic differentiation in DOP. It is suggested that miR-1263 regulates apoptosis and osteogenic differentiation of BMSCs through the Mob1/Hippo signaling pathway. In addition, Li et al. [103] explored the effect of hBMSC-Exo-miR-186 on postmenopausal OP. Mob1, a cofactor that regulates YAP, is a potential target of miR-186 [104]. This study found that hBMSC-Exos and miR-186 mimics promoted the expression of YAP, while miR-186 inhibitors decreased the expression of YAP and Mob1. Further studies revealed that hBMSC-Exos or miR-186 mimics increased bone volume in OVX rats, while miR-186 inhibitors decreased bone volume. It suggests that hBMSC-Exos promotes bone formation in OVX rats through the Hippo signaling pathway by transferring the intrinsic miR-186.

The above studies illustrate the positive role of the Hippo signaling pathway in the treatment of OP by MSC-EVs. Notably, the Hippo signaling pathway forms a sophisticated molecular regulatory network around the upstream protein kinase MST1/2 and the downstream effector molecule YAP/TAZ, which have important roles in maintaining normal physiological functions of bone. However, there is no consensus on the complex regulatory mechanisms of MST1/2 and YAP/TAZ. Further exploration of the series of molecular events of MST1/2, YAP/TAZ and their upstream and downstream transcription factors is necessary to provide new ideas for related studies of OP.

#### 4.1.3. PI3K/Akt Signaling Mediates MSC-EVs Regulating Bone Formation in OP

PI3K/Akt signaling is closely associated with cell proliferation, differentiation, and apoptosis in many tissues [105]. Upon stimulation by growth factors, PI3K is activated to produce phosphatidylinositol trisphosphate, which binds to Akt to translocate Akt from the cytoplasm to the cell membrane while undergoing a conformational change. Subsequently, Akt is activated and further activates downstream target genes to participate in cell growth and differentiation [106]. Previous studies have found that the PI3K/Akt signaling pathway plays an important role in bone formation by regulating the potential and direction of early BMSC differentiation [107]. Lu et al. [108] analyzed the changes in mRNA expression profiles in bone tissue of OVX mice treated with or without MSC-EVs by the next-generation sequencing (NGS) technique to identify the key intrinsic cargoes and the key signaling pathways involved in the treatment of OP with MSC-EVs. This study found that MSC-EVs may exert their therapeutic effects on OP by upregulating extracellular matrix-associated gene expression and activating the PI3K/Akt signaling pathway through the intrinsic miRNAs, including miR-21, miR-29, and miR-221. However, the specific mechanism still needs to be further explored. Furthermore, Zhang et al. [109] explored in depth the possible regulatory mechanism of miR-22-3p loaded by MSC-EVs on bone formation in OP by loss and gain of function experiments. The results revealed that overexpression of miR-22-3p encapsulated in MSC-EVs could upregulate the expression of osteogenic genes such as Runx2, osteocalcin (OCN), and osteopontin (OPN) and enhance ALP activity and matrix mineralization in BMSCs by directly targeting alpha-ketoglutarate-dependent dioxygenase FTO (FTO), whereas miR-22-3p inhibitor could repress osteogenic differentiation of BMSCs, which may be closely related to the MYC/PI3K/Akt pathway. Further studies showed that FTO silencing could partially reverse the inhibition of osteogenic differentiation caused by miR-22-3p inhibitors [109]. It suggests that MSC-EV-miR-22-3p can promote osteogenic differentiation via the MYC/PI3K/Akt pathway.

In summary, multiple miRNAs encapsulated in MSC-EVs, including miR-21, miR-29, miR-221, and miR-22-3p, can promote osteogenic differentiation in OP via the PI3K/Akt signaling pathway. However, EV-miRNAs have a complicated readout in both physiological and pathophysiological states. For the detected miRNAs, the conclusions obtained are usually unreliable due to the presence of most EV-miRNAs universally expressed making it difficult to trace the specific tissues in which they function. Therefore, tissue-specific miRNAs should be considered to provide a clear demonstration of the specific roles of certain miRNAs in the future.

#### 4.1.4. NF-κB Signaling Mediates MSC-EVs Regulating Bone Formation in OP

Since the role of NF-κB in bone was first identified in the mid-1990s, the role of NF-κB signaling in bone development and bone disease has been extensively studied [110]. NF-κB is a transcription factor present in mammalian cells that rapidly responds to various stimuli to regulate downstream signaling cascades, thus participating in the inflammatory response, immune response, cell survival, and apoptosis [111]. NF-κB signaling has been found to be involved in regulating the proliferation, differentiation, and apoptosis of osteoblasts, osteoclasts, osteocytes, and chondrocytes, thus playing an important role in the initiation and development of OP [112]. Li et al. [113] determined the role of hBMSC-EVs in osteogenic differentiation by overexpressing or knocking down KLF2, WWP1, and miR-15b in hBMSCs. The team found that upregulation of miR-15b or KLF2 or downregulation of WWP1 or NF-κB increased the expression of osteogenic differentiation-related genes and promoted ALP activity and stromal mineralization in hBMSCs. Further studies revealed that miR-15b directly targets WWP1 to attenuate KLF2 ubiquitinated degradation and inhibit NF-κB activity. BMSC-EV-miR-15b increased bone volume and bone trabeculae number in OVX rats, while EV-miR-15b inhibitor partially reversed the increase in bone mass in OP mice caused by EV-miR-15b, which was closely associated with NF-κB signaling. The above results suggest that miR-15b loaded by BMSC-EVs promotes osteogenic differentiation by inhibiting the target gene WWP1-mediated KLF2 ubiquitination and NF-κB signaling pathway. In addition, lysine demethylase 5A (KDM5A) is enriched in the suppressor of cytokine signaling 1 (SOCS1) promoter region [114], which has been shown to be involved in the etiology of OP [115]. Previous studies reported that SOCS1, as an upstream regulator of NF-κB activation, can directly bind to NF-κB-p65 to inhibit NF-κB activation, ultimately participating in the regulation of osteogenic differentiation [116]. Zhang et al. [117] collected bone marrow specimens from OP patients undergoing total hip arthroplasty (THA) and non-OP patients to detect the expression of BMSC-EV-miRNAs and performed further functional analysis in an OP mouse model. They found that BMSC-EV-miR-29b-3p specifically targets KDM5A and represses H3K4me3 and H3K27ac in the SOCS1 promoter region, thereby inhibiting their expression and downstream NF-κB signaling to promote osteogenic differentiation of BMSCs [117]. Notably, other studies have reported that miR-29b-3p can reduce the expression of multiple genes related to the regulation of bone formation, such as histone deacetylase 4 (HDAC4), transforming growth factor-β3 (TGFb3), activator receptor type 2A (ACVR2A), β-linked protein interaction protein 1 (CTNNBIP1), and dual-specificity protein phosphatase 2 (DUSP2) [118]. The above contradictory results may be due to different cellular microenvironments leading to functional differences in miR-29b-3p, or it may also be that miR-29b-3p is differentially expressed at different stages of bone formation. Further studies are needed to clarify the specific role of miR-29b-3p in bone formation.

The above study showed that MSC-EVs inhibit NF-κB signaling pathway by delivering intrinsic miRNAs, such as miR-15b and miR-29b-3p, to promote osteogenic differentiation in OP. However, the complex composition of the cargoes contained within MSC-EVs and the concentration of MSC-EVs administered varies considerably in different experiments, which leads to possible differences in the therapeutic effects of MSC-EVs in the OP. While further exploring the potential mechanisms of active ingredients within MSC-EVs to regulate osteogenic differentiation, attention needs to be paid to the dosing regimen of MSC-EVs to achieve optimal OP therapeutic effects.

#### 4.1.5. SATB2 Signaling Mediates MSC-EVs Regulating Bone Formation in OP

SATB2, a member of the SATB family, is a protein that binds to nuclear matrix regions to regulate gene transcription and chromatin reorganization. SATB2 has been reported to play an important role in a variety of physiological and pathological processes, including nervous system development, tumorigenesis, and bone metabolism [119]. Recent studies have shown that SATB2 has a regulatory role in osteogenic gene expression patterns and directed osteogenic differentiation [120]. In addition, SATB2 has been suggested to be a specific immunohistochemical biomarker of osteoblast differentiation [121]. However, the specific mechanism of action of SATB2-mediated MSC-EVs in OP therapy is not clear. Xu et al. [81] found that compared with young rats, the expression level of BMSC-EV-miR-31a-5p, which promotes bone resorption, was significantly higher in aged rats, whereas inhibition of miR-31a-5p impedes bone loss in aged rats. It was further found that SATB2 is a target gene of miR-31a-5p. Si-SATB2 treatment was able to partially reverse the osteogenic differentiation of BMSCs inhibited by BMSC-Exo-miR-31a-5p, suggesting that BMSC-Exo-miR-31a-5p inhibits osteogenic differentiation of BMSCs in OP by negatively regulating SATB2 expression. Therefore, the miR-31a-5p-SATB2 axis plays a crucial role in the osteogenic differentiation of BMSCs with osteoporotic bone loss during aging. In addition, lncRNAs are emerging as novel regulators of the osteogenesis process in MSCs [122]. Knockdown of lncRNA metastasis-associated lung adenocarcinoma transcript 1 (MALAT1) reversed RANKL activator-induced growth inhibition in osteoblast cell line (hFOB1.19) [123]. Moreover, Yang et al. [124] found that BMSC-Exo-lncRNA MALAT1 promoted osteoblast activity by upregulating SATB2, while silencing SATB2 inhibited osteoblast activity. Further studies revealed that miR-34c inhibited the osteogenic effect of BMSC-Exo-lncRNA MALAT1 in OVX mice, while SATB2 reversed the effect of miR-34c. It indicates that LncRNA MALAT1 shuttled by BMSC-Exos alleviates OP by enhancing bone formation through miR-34c/SATB2 axis. Notably, bioinformatic analysis has been performed to determine that miR-34c can bind to MALAT1 and SATB2, respectively [124]. However, further experiments are needed to characterize whether miR-34c and MALAT1 may form a competing endogenous RNA network and to validate the expression patterns of MALAT1, miR-34c and SATB2 in clinical samples.

In summary, MSC-EVs-encapsulated miRNAs or lncRNAs promotes osteogenic differentiation by regulating SATB2 signaling, providing a broader understanding of the pathogenesis of OP and offering a potential therapeutic strategy for OP treatment.

### 4.2. Regulation of Bone Resorption by MSC-EVs in OP

Osteoclasts, the main effector cells of bone resorption, play a crucial role in skeletal development and in the pathogenesis of bone diseases [125]. Osteoclasts are derived from the monocyte/macrophage lineage, which can enhance bone resorption activity by secreting acidic substances and proteases. The relative enhancement of osteoclast activity is the key cause of OP [126]. Previous studies found that BMSC-EVs transfer intrinsic miR-143/145 to osteoblasts to trigger osteoclast activity and differentiation by targeting Cd226 and Srgap2 [127]. Furthermore, Chen et al. [128] found that osteoclast-associated genes such as Trap, MMP9, and Ctsk were repressed in OP mice treated with human urine-derived stem cells (USCs)-Exos and confirmed that collagen triple-helix repeat loaded in Exos-containing 1 (CTHRC1) and osteoprotegerin (OPG) proteins in Exos inhibited osteoclastogenesis. RANKL, as a member of the RANK/RANKL pathway, is essential in regulating bone remodeling [129]. RANKL secreted by osteoblasts binds to the receptor RANK on the surface of osteoclasts and their precursor cells to promote osteoclast survival and stimulate osteoblast maturation, proliferation, and differentiation [130]. Ren et al. [131] found that Exos derived from adipose-derived stem cells (ADSCs) inhibited RANKL expression at the mRNA and protein levels and reduced the RANKL/OPG ratio, thereby improving osteocyte-mediated osteoclastogenesis in vitro. Moreover, Hu et al. [5] revealed that the potently pro-osteogenic protein, CLEC11A (C-type lectin domain family 11, member A) encapsulated by HucMSC-EVs inhibits osteoclastogenesis by suppressing RANKL expression, thereby alleviating OP. OPG is a soluble protein that binds to RANKL to prevent RANKL from activating RANK. Lee et al. [132] found that ADSC-EVs significantly inhibited the differentiation of macrophages to osteoclasts and promoted the migration of BMSCs, which in turn attenuated bone loss in OP mice. However, OPG-deficient ADSC-EVs did not exhibit an anti-osteoclastogenic effect. Further studies revealed that OPG in ADSC-EVs was involved in inhibiting osteoclast differentiation and reducing the expression of genes related to bone resorption [132]. The above studies suggest that OPG/RANKL/RANK signaling may be involved in cell-free therapy of OP by ADSC-EVs. Furthermore, Xiao et al. [133] constructed hindlimb unloading-induced DOP mouse models to compare cyclic mechanical stretch (CMS)-treated BMSC-Exos (CMS-Exos) and normal static-cultured BMSC-Exos (static-Exos) in DOP. The results showed that although both CMS-Exos and static-Exos partially rescued mechanical unloading-induced OP, the CMS-Exo group exhibited a more significant therapeutic effect. Mechanistically, CMS-Exos impairs osteoclast differentiation by inhibiting the activity of the RANKL-induced NF-κB signaling pathway and ameliorates bone loss induced by mechanical unloading in a hindlimb unloading DOP mouse model. In addition, another study found that miR-27a released from MSC-EVs can inhibit osteoclast differentiation through the DKK2/Wnt/β-catenin signaling pathway [93]. In contrast, one study revealed that aged rat BMSC-Exo-miR-31a-5p positively regulates osteoclastogenesis by targeting RhoA signaling, thereby exacerbating OP [81].

**Figure 3:**
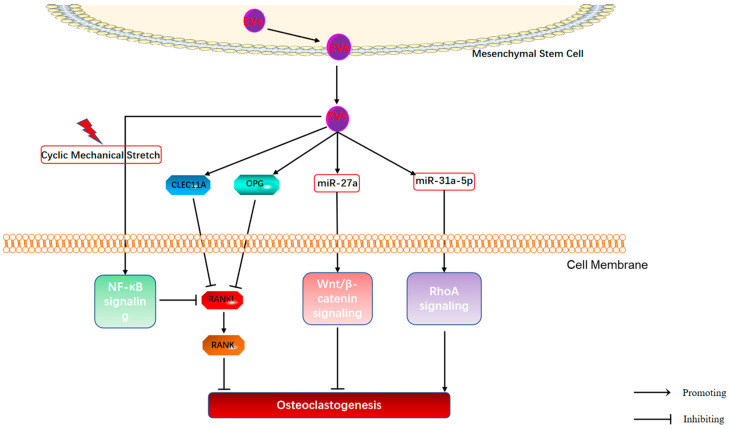
Mechanisms of MSC-EVs regulating the osteoclastogenesis in OP.

In summary, MSC-EVs can inhibit osteoclastogenesis via OPG/RANKL/RANK, NF-κB, and Wnt/β-catenin signaling pathways, whereas the RhoA signaling pathway exerted the opposite effect on osteoclastogenesis, depending on whether the parental cells of EVs were derived from normal or senescent MSCs (As shown in Figure 3). Notably, CMS-Exos treatment, but not static-Exos, improves cortical bone loss in OP [133]. It may be that mechanical stimulation alters the intrinsic cargo composition of EVs to confer stronger anti-OP activity to EVs, indicating that the therapeutic effect of EVs can be enhanced by modulating the parental cell microenvironment.

### 4.3. Regulation of Bone Angiogenesis by MSC-EVs in OP

Angiogenesis is of great importance for bone reconstruction. On the one hand, it provides bone tissue with the required oxygen and nutrients; on the other hand, it provides calcium and phosphate to facilitate bone mineralization. Impaired angiogenesis predisposes to impaired bone regeneration, which is one of the major causes of OP [134]. An important vascular subtype (type-H vessels) was found to regulate the growth of the bone vascular system, recruit osteogenic progenitor cells, and integrate osteogenesis and angiogenesis [135]. Stimulation of type-H vessel formation can partially rescue bone loss [136]. Cumulative studies have found that MSC-EVs are enriched in platelet-derived growth factor, epidermal growth factor, fibroblast growth factor, and NF-κB signaling axis-related proteins. NF-κB is a key mediator of endothelial cell angiogenesis induced by MSC-EVs. It suggests that MSC-EVs contain many pro-angiogenic paracrine effector molecules [137]. Qi et al. [39] first reported that iMSC-Exos can promote bone regeneration by enhancing angiogenesis and osteogenesis in OP rats, indicating that iMSC-Exos has the potential to promote angiogenesis in OP. Subsequently, an Exos delivery system based on iPSC-MSC-Exos was developed to silence the SHN3 gene in osteoblasts and promote the production of the proangiogenic factor slit guidance ligand 3 (SLIT3), which increases type-H vessel formation and thus alleviates OP [73]. In addition, Lu et al. [45] found that BMSC-Exos could be taken up by human umbilical vein endothelial cells (HUVECs) and promoted the proliferation, migration, and tube formation of HUVECs, ultimately promoting angiogenesis and improving OP symptoms in OP mouse models. Further analysis revealed that miR-29a loaded in BMSC-Exos promotes angiogenesis by directly targeting inhibition of VASH1. In addition, Behera et al. [138] identified a bone-specific lncRNA H19 in BMSC-Exos by sequencing analysis and subsequently evaluated the effect of BMSC-Exos on angiogenesis in an immunodeficient cystathionine β-synthase (CBS)-heterozygous mouse model. This study revealed that lncRNA H19 in BMSC-Exos targeted repression of miR-106 expression through molecular sponge action and negatively regulated the expression of angiogenic factor Angpt1. Subsequently, reduction of Angpt1 in endothelial cells further activates Tie2-NO signaling and ultimately significantly promotes angiogenesis in CBS-heterozygous mice.

**Figure 4:**
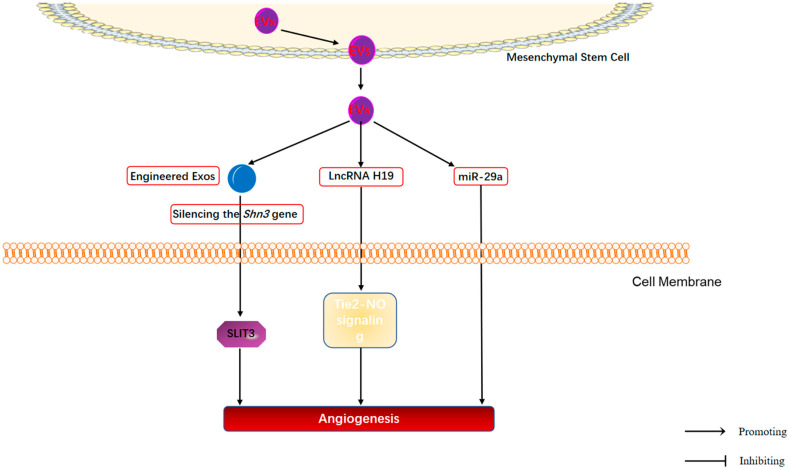
Mechanisms of MSC-EVs regulating the angiogenesis in OP.

In summary, MSC-EVs demonstrated their great potential to promote angiogenesis in OP (As shown in Figure 4). However, the specific mechanisms involved are still unclear and further studies are needed. Furthermore, although miR-29a and lncRNA H19 loaded in MSC-EVs have been found to play a crucial role in regulating angiogenesis, other bioactive molecules encapsulated in MSC-EVs may also be involved in the regulation of angiogenesis by MSC-EVs in OP. Further screening of other potential key components in MSC-EVs to regulate angiogenesis is required in OP.

### 4.4. Regulation of Bone Immunity by MSC-EVs in OP

Previous studies have shown that OP is closely related to the immune inflammatory system. The activation of the immune system and the release of inflammatory factors play an important role in the initiation and development of OP [139]. The activation and proliferation of immune inflammatory cells, mainly T lymphocytes, leads to the release of inflammatory factors such as interleukin-1β (IL-1β) and TNF-α and promotes osteoclast formation, which exacerbates bone resorption [140]. MSC-EVs have been shown to have a wide range of immunomodulatory effects, including suppressing T lymphocyte activity and promoting apoptosis, inhibiting inflammatory factor secretion, reducing neutrophil aggregation, inducing conversion of Th1 to Th2 cell type, and reducing the potential for T cell differentiation to Th17 cell type [141,142]. Therefore, MSC-EVs may play an immunomodulatory role in the treatment of OP. Zhang et al. [143] explored the effects of ADSC-Exos on cellular and animal models of diabetic OP constructed by high glucose exposure and streptozotocin injection. It was found that ADSC-Exos inhibited high glucose-induced secretion of secretion of IL-1β and IL-18 by osteoclasts, decreased the expression and activation of NLRP3 inflammasome-associated proteins (included pro-caspase-1, sensor protein NLRP3, and adaptor protein ASC) and restored bone loss in diabetic osteoporotic rats. Mechanistically, ADSC-Exos inhibited the activation of NLRP3 inflammasome in osteoclasts and reduced the production of inflammatory mediators including TNF-α, IL-6, PGE2, and NO, which in turn reduced bone resorption and restored bone loss [143], suggesting that ADSC-Exos may be a potential cell-free therapeutic strategy for diabetic bone loss. In addition, studies have reported miR-146a as a core mediator in the anti-inflammatory action of Exos [144]. To investigate the role of MSC-Exo-miR-146a in the treatment of diabetic OP, Zhang et al. [82] extracted miR-146a-enriched Exos from ADSCs overexpressing miR-146a to explore its protective effect against osteoclast inflammation. The results revealed that miR-146a-Exos had more potent inhibitory effect than Exos/vector-Exos in reducing the expression and secretion of pro-inflammatory cytokines such as TNF-α, IL-18, and IL-1β in osteoclasts and bone resorption in vivo and vitro. Moreover, the anti-inflammation effect of miR-146a has been reported in other cellular and animal models, and Exos with overexpressed miR-146a was able to magnify the anti-inflammation effect. Overall, ADSC-Exo-miR-146a could effectively inhibit the expression of pro-inflammatory cytokines, secreted by osteoclasts induced by high glucose, induce the inflammasome inactivation, inhibit bone resorption, and ultimately restore bone loss in diabetic osteoporotic rats, indicating that ADSC-Exo-miR-146a can effectively inhibit the inflammatory response of osteoclasts and provide a potential strategy for the treatment of diabetic OP.

In summary, MSC-EVs can effectively inhibit the expression of pro-inflammatory cytokines produced by osteoclasts and the activation of inflammasome, thereby alleviating the development of OP. Interestingly, miR-146a, a well-known anti-inflammatory miRNA, targets effector molecules in different cell types. miR-146a is predicted to form base pairs with pro-inflammatory cytokines (e.g., TNF-α, IL-18, and IL-1β) in osteoblasts at the 3′UTR region of TRAF6 and IRAK1 [145]. However, whether miR-146a can act directly or through other unknown regulatory factors on TNF-α, IL-18, and IL-1β in osteoblasts needs to be further investigated.

## 5. Conclusions and Perspectives 

This paper comprehensively describes the recent advances in the application of specific targeted therapy of MSC-EVs in OP. In addition, this paper systematically details the potential mechanisms of MSC-EVs in OP from four aspects, including promotion of osteogenic differentiation, inhibition of osteoclast function, promotion of angiogenesis, and immunomodulation. MSC-EVs can regulate key signals for bone formation and bone resorption by delivering intrinsic miRNAs and other bioactive substances, including Wnt/β-catenin, Hippo, PI3K/Akt, NF-κB, SATB2, RANKL, and RhoA (as shown in Table 1). It was shown that MSC-EVs can also promote angiogenesis through the lncRNAH19-Angpt1-Tie2/NO signaling pathway. Furthermore, MSC-EVs were found to effectively inhibit the expression of pro-inflammatory cytokines, such as TNF-α, IL-18, and IL-1β, induce NLRP3 inflammasome inactivation, which in turn inhibits bone resorption and alleviates OP. The above studies suggest that MSC-EVs have shown good therapeutic effects in the prevention and treatment of OP, which may provide a new idea for OP treatment-related research.

Although MSC-EVs show positive promise in the treatment of OP models in vivo and in vitro. However, there are still many issues that need to be addressed. (1) The application of MSC-EVs in OP is at a preliminary stage. It is needed to unify the issues of extraction, isolation, purification, mass production methods, and therapeutic doses of MSC-EVs. Therefore, further studies are needed to determine the optimal therapeutic dose, injection interval, appropriate injection route, isolation method, and optimization of EVs culture conditions for OP treatment in order to achieve optimal therapeutic effects and minimal adverse effects [146]; (2) since many miRNAs are encapsulated in EVs and different miRNAs may interact with each other to form complex networks of regulation, the potential mechanisms of EVs in OP are still not fully understood. In addition to miRNA, EVs contain many proteins, lipids, and other types of nucleic acids such as lncRNAs, circRNAs, and mRNAs. Not only do the regulatory mechanisms in the relief of OP by EVs-derived miRNAs need to be further elucidated, but also the role of other intrinsic molecules of EVs in the relief of OP needs to be further explored; (3) although endogenous cargoes of MSC-EVs can be modified and tailored, the optimal pretreatment regimen to enhance their therapeutic potential is largely unknown due to complex mechanisms of interaction between cargoes, such as targeted repression of miRNAs and mRNAs, and the various epistatic modifications that may be present [147]. (4) The last but not the least problem is that the administration concentration and dosage of exosomes in vivo have not been unified. EVs at different concentrations were not used to explore the dose-effects, and even the administration concentration and dose of EVs were not described in detail in most of the experiments. Different doses of MSC-EVs may have different therapeutic effects in the treatment of OP. Although the potential role of MSC-EVs in OP models has been demonstrated, the effective application of MSC-EVs in clinical treatment still faces many challenges, and further clinical trials are needed.

## Figures and Tables

**Table 1:** Mechanisms of MSC-EVs in OP.

Source	Cargoes/Stimulation	Signaling	Function	References
Regulation of Bone Formation				
BMSC-Exos	miR-196a	Wnt/β-catenin	Promoting Bone Formation	[91]
BMSC-EVs	GPNMB	Wnt/β-catenin	Promoting Bone Formation	[95]
MSC-EVs	miR-27a	Wnt/β-Catenin	Promoting Bone Formation	[93]
BMSC-Exos	miR-424-5p	Wnt/β-catenin	Inhibiting Bone Formation	[98]
HESC-EVs	-	Wnt/β-catenin	Promoting Bone Formation	[90]
BMSC-Exos	miR-186	Hippo	Promoting Bone Formation	[103]
HUCMSC-Exos	miR-1263	Hippo	Promoting Bone Formation	[102]
BMSC-Exos	miR-22-3p	PI3K/AKT	Promoting Bone Formation	[109]
MSC-EVs	miR-21, miR-29, miR-221	PI3K/AKT	Promoting Bone Formation	[108]
BMSC-EVs	miR-29b-3p	NF-κB	Inhibiting Bone Formation	[117]
BMSC-EVs	miR-15b	NF-κB	Inhibiting Bone Formation	[113]
BMSC-Exos	lncRNA MALAT1	SATB2	Promoting Bone Formation	[124]
BMSC-Exos	miR-31a-5p	SATB2	Inhibiting Bone Formation	[81]
Regulation of Bone Resorption				
ADSC-Exos	-	-	Inhibiting Bone Resorption	[131]
ADSC-EVs	miR-21-5p, OPG	-	Inhibiting Bone Resorption	[132]
BMSC-Exos	Mechanical Stretch	NF-κB	Inhibiting Bone Resorption	[133]
BMSC-Exos	miR-31a-5p	RhoA	Promoting Bone Resorption	[81]
MSC-EVs	miR-27a	Wnt/β-Catenin	Inhibiting Bone Resorption	[93]
HUCMSC-EVs	CLEC11A	-	Inhibiting Bone Resorption	[5]
Regulation of Bone Angiogenesis				
HIPSC-Exos	Shn3	-	Promoting Angiogenesis	[73]
BMSC-Exos	miR-29a	-	Promoting Angiogenesis	[45]
BMSC-Exos	lncRNA-H19	Angpt1/Tie2-NO	Promoting Angiogenesis	[138]
HIPSC-Exos	-	-	Promoting Angiogenesis	[39]
MSC-Exos	-	NF-κB	Promoting Angiogenesis	[137]
Regulation of Bone Immunity				
ADSC-Exos	-	-	Inhibiting inflammatory response	[143]
ADSC-Exos	miR-146a	-	Inhibiting inflammatory response	[82]

## Data Availability

Not applicable.
